# Geographic Variation in Advertisement Calls in a Tree Frog Species: Gene Flow and Selection Hypotheses

**DOI:** 10.1371/journal.pone.0023297

**Published:** 2011-08-17

**Authors:** Yikweon Jang, Eun Hye Hahm, Hyun-Jung Lee, Soyeon Park, Yong-Jin Won, Jae C. Choe

**Affiliations:** Division of EcoScience, Ewha Womans University, Seoul, Republic of Korea; University of Maribor, Slovenia

## Abstract

**Background:**

In a species with a large distribution relative to its dispersal capacity, geographic variation in traits may be explained by gene flow, selection, or the combined effects of both. Studies of genetic diversity using neutral molecular markers show that patterns of isolation by distance (IBD) or barrier effect may be evident for geographic variation at the molecular level in amphibian species. However, selective factors such as habitat, predator, or interspecific interactions may be critical for geographic variation in sexual traits. We studied geographic variation in advertisement calls in the tree frog *Hyla japonica* to understand patterns of variation in these traits across Korea and provide clues about the underlying forces for variation.

**Methodology:**

We recorded calls of *H. japonica* in three breeding seasons from 17 localities including localities in remote Jeju Island. Call characters analyzed were note repetition rate (NRR), note duration (ND), and dominant frequency (DF), along with snout-to-vent length.

**Results:**

The findings of a barrier effect on DF and a longitudinal variation in NRR seemed to suggest that an open sea between the mainland and Jeju Island and mountain ranges dominated by the north-south Taebaek Mountains were related to geographic variation in call characters. Furthermore, there was a pattern of IBD in mitochondrial DNA sequences. However, no comparable pattern of IBD was found between geographic distance and call characters. We also failed to detect any effects of habitat or interspecific interaction on call characters.

**Conclusions:**

Geographic variations in call characters as well as mitochondrial DNA sequences were largely stratified by geographic factors such as distance and barriers in Korean populations of *H. japoinca*. Although we did not detect effects of habitat or interspecific interaction, some other selective factors such as sexual selection might still be operating on call characters in conjunction with restricted gene flow.

## Introduction

When a species has a wide distributional range relative to its dispersal capacity, geographic variation in a trait may be explained by the balance of gene flow and selection [1], [2], [3]. The level of gene flow is at maximum between adjacent populations and decreases with increase in geographic distance. The negative correlation between genetic similarity and geographic distance between populations is known as isolation by distance (IBD) [4], [5], [6]. In addition, values of a trait are more dissimilar across geographical barriers, independent of geographic distance. It is generally accepted that organisms with low vagility would have higher levels of IBD or stronger effects of geographic barriers on traits [7]. When gene flow between populations is prevented due to geographic distance or a barrier, populations may rapidly respond to other evolutionary processes such as selection or genetic drift, forces which vary across a species' geographic range and may result in geographic variation in traits.

Selection, which varies temporally and spatially, is also an important evolutionary force for broad-scale geographic variation in a trait. Demonstration of a selective factor for geographic variation in a trait requires identification of the factor and differential response of the trait to the factor. When a trait is under selection, a predicted pattern of the trait may be discontinuous between different states of a selective regime. In addition to gene flow and selection, genetic drift might play a role in shaping geographic distribution in traits. However, the effect of genetic drift on phenotypic traits is difficult to study, because its effect may be weak in large populations [8] or neutral with no selective difference between genotypes [9], [10]. Besides these evolutionary processes, population genetic structures and historical demographic changes could play an important role in geographic variation in traits [11]. For example, when a species has recently undergone rapid range expansion, populations have not much time to respond to local differences in selective regimes [1].

Many studies of genetic diversity have concluded that gene flow is critical for geographic variation in amphibians [12]. That is, the pattern of IBD [7], [13], [14], [15], [16] and barrier effect [17], [18], [19], [20], [21] are evident for geographic variation of genetic diversity in most amphibian species studied. Although the results of genetic variation using seemingly neutral molecular markers (mostly mitochondrial and microsatellite DNAs) are valuable for understanding patterns of genetic structure and phylogeography among populations in a species, findings of these results may not be directly extrapolated to traits that are regularly and strongly subject to selection.

In most amphibian taxa, males produce advertisement calls that are attractive to conspecific females [22]. Possible selective factors for a sexually selected trait include habitat, predators, competitors, or ecological or reproductive interactions between closely related species. For some frog species there is evidence that geographic variation in male call characters is generally concordant with the pattern of gene flow [18], [23], [24]. However, male advertisement calls and female preferences in amphibians are often subject to sexual selection, leading to reproductive isolation among populations [25]. Because the development of frog calls is mostly determined at genetic levels, frog calls seem to be less influenced by broad-scale habitat characteristics [26], [27]. However, in two allopatric genetic lineages of *Physalaemus pustulosus* in central Costa Rica, geographic variation in population structure and advertisement calls were attributed to habitat difference in conjunction with lack of movement between lineages [13], [23], [28]. In addition, there is a growing number of examples in which geographic variation in both advertisement calls and female preferences may be influenced by interspecific interaction between closely related frog species [29], [30], [31].

We studied geographic variation in advertisement call characters of the tree frog *Hyla japonica* in the Republic of Korea to understand evolutionary forces shaping the variation. The genetic diversity using mitochondrial *COI* gene sequences was also estimated to understand the population structure of *H. japonica*. We specifically tested two hypotheses about geographic variation in call characters. In the gene flow hypothesis, we predicted a pattern of IBD or the barrier effect on geographic variation in a call character as well as genetic differentiation. In the selection hypothesis, we predicted a discontinuous pattern on geographic variation in call characters relative to different states of habitat and degree of population overlap (sympatry versus allopatry).

## Methods

### Ethical treatment of animals

The Institutional Animal Care and Use Committee, which oversees animal experimentation at Ewha University, was not established when this study was conducted. However, we treated our study subject, *Hyla japonica*, in strict accordance with the recommendations of the Animal Behaviour Society [32]. *H. japonica* is not listed as an endangered species and, in fact, is one of the most abundant frog species in Korea.

### Study species

**Figure 1:**
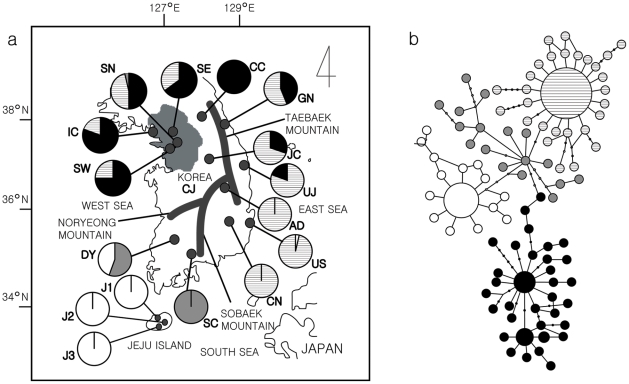
Recording localities and haplotype network sampled for advertisement calls of *H. japonica*. The left panel (a) shows recording localities sampled for advertisement calls of *H. japonica*. A pie graph represents frequencies of the four *COI* haplogroups in each locality. Thick lines indicate major mountain ranges in the Republic of Korea. Advertisement calls of *H. japonica* were recorded in 13 allopatric localities: J1, J2, J3, SC, DY, US, CN, AD, CJ, UJ, GN, JC, and CC. *H. japonica* is sympatric with *H. suweonensis* in or around the Gyeonggi Province, which is the shaded region. Thus, these two species can be potentially found together in SW, SN, IC and SE. See Table 1 for locality abbreviation and the number of recorded advertisement calls for each locality. The population genetic study was conducted for all but one locality, CJ. We found 95 unique haplotypes of *COI* among all 339 individual tree frogs from 16 localities in Korea. In the haplotype network (b), the size of a circle is proportional to the frequency of its haplotype. The different fill patterns of pies on the map correspond to those of the haplotype network.

*Hyla japonica*, one of the most common frog species in Korea, is distributed widely in East Asia, ranging from Manchuria to southern China, and from Mongolia to Japan. From May to August in Korea, *H. japonica* aggregates to breed in places that hold water, such as rice paddies and wetlands. Males produce species-specific calls that attract conspecific receptive females. In addition to *H. japonica*, there is another tree frog species, *H. suweonensis*, which is endemic to Korea. *H. suweonensis* is known to be distributed in small areas in the Gyeonggi Province (Fig. 1) [33]. The two tree frog species are not clearly distinguishable based on morphological traits. However, calls of *H. suweonensis* have longer note duration and longer note interval than those of *H. japonica*. The genetic differentiation between the two is similar to inter-species variation in other amphibians, based on studies using allozymes and mitochondrial DNA [34], [35], [36]. In addition to *H. suweonensis*, *H. japonica* is sympatric with the black-spotted pond frog (*Rana nigromaculata*), the Korean golden frog (*R. plandyi chosenica*), the bullfrog (*Lithobates catesbeianus*), and the narrow-mouth frog (*Kaloula borealis*) in Korea. The breeding seasons of these species largely overlap, but the advertisement calls are species-specific.

### Study sites

We recorded advertisement calls of *H. japonica* from 17 localities in the Republic of Korea. Geographic information and abbreviations of these localities are found in Table 1 and Fig. 1. We used a GPS receiver (GARMIN GPSMAP 60CSx Portable Navigator, Kansas, USA) to record latitude, longitude and elevation. The habitats of *H. japonica* may be classified into two types in this study: rice paddies (CC, IC, SW, UH, CJ, US, CN, DY, SC, AD, JC and GN) and non-rice paddies (SE, SN, J1, J2, and J3). In Korea, rice paddies are temporary wetlands where rice seedlings grow from May to October every year. They contain water usually between May and August and provide habitats for frogs and aquatic insects. Rice paddies are basically open, flat fields without any obstacles to sound transmission. By contrast, the topography of non-rice paddy habitats is generally more complex. Two localities on Jeju Island (J2 and J3) are surrounded by trees and shrubs. J1 is in a pond of a Buddhist temple and is in the vicinity of low buildings and other obstacles. SE is in a bush near a stream. In addition to the quality of sound transmission, the two habitat types may differ in pesticide use. Pesticides are applied regularly during the rice-growing season in rice paddy localities, whereas use of pesticides is unlikely in non-rice paddy localities. However, J1 might be exposed to pesticide use from nearby lawn or crops. We classified SN as a non-rice paddy habitat, even though this locality contained rice paddies. This locality was surrounded by pine and oak trees that are typical of deciduous forests in central Korea. There were four rice paddies, arranged like steps vertically, ranging in size from about 10 to 35 m^2^. Rice paddies and the pond were maintained not for crop production but to provide habitat for fireflies. Thus, agricultural chemicals were strictly prohibited in this wetland. Although rice seedlings were planted at the beginning of early June, weeds become dominant in the rice paddies by late August.

**Table 1:** Recording localities for *H. japonica*.

Locality	Abbr.	*n_1_*	*n_2_*	Recording date	Latitude	Longitude	Elevation	Habitat	Patry
Buphwa-Sa	J1	10	10	July 9–13, 2007	33°15.40′N	126°27.36′E	140 m	N	A
Seogipo Forest	J2	8	12	July 9–13, 2007	33°18.30′N	126°27.40′E	630 m	N	A
1100 Wetland	J3	24	22	July 9–13, 2007	33°21.16′N	126°27.53′E	1100 m	N	A
Suncheon	SC	24	12	June 29–30, 2008	34°53.29′N	127°30.55′E	9 m	R	A
Damyang	DY	27	27	July 1, 2008	35°18. 98′N	126°54.68′E	213 m	R	A
Ulsan	US	21	28	June 22–24, 2008	35°30.47′N	129°08.97′E	93 m	R	A
Changnyung	CN	19	28	June 25–27, 2008	35°34.50′N	128°26.23′E	27 m	R	A
Andong	AD	21	21	June 24–26, 2009	36°33.70′N	128°31.38′E	84 m	R	A
Chungju	CJ	21	0	June 22–26, 2007	36°36.23′N	127°29.00′E	54 m	R	A
Uljin	UJ	18	26	July 15–17, 2008	37°01.13′N	129°22.92′E	82 m	R	A
Jecheon	JC	20	23	June 27–28, 2009	37°04.34′N	128°10.66′E	166 m	R	A
Suwon	SW	6	8	July 18, 2008	37°16.12′N	126°59.38′E	70 m	R	S
Sungnam	SN	22	28	July 10–11, 2008	37°24.22′N	127°08.11′E	102 m	N	S
Incheon	IC	22	26	July 2–4, 2007	37°36.36′N	126°27.50′E	10 m	R	S
Seoul	SE	18	20	July 20, 2007	37°39.13′N	126°56.56′E	80 m	N	S
Gangneung	GN	25	25	June 30–July 1, 2009	37°46.46′N	128°46.06′E	92 m	R	A
Chuncheon	CC	18	23	July 23–25, 2007	37°59.24′N	127°44.24′E	210 m	R	A

*n_1_* is the number of calls recorded, and *n_2_* is the number of samples for DNA analysis.

Habitat was classified as rice paddy (R) or non-rice paddy (N). Patry was classified as sympatry (S) if a locality was in the Gyeonggi Province or as allopatry (A) if a locality was outside the Gyeonggi Province [87].

The Korean Peninsula is mostly mountainous and not arable. Lowlands lie along the coasts, particularly along the West Sea and South Sea. Along the East/Japanese Sea, the Taebaek Mountain Range runs north and south as a continuous ridge with an average height of about 1,000 m (Fig. 1). The eastern slopes of the mountain range fall steeply into the sea, but the western slopes are more gradual. Split off from the Taebaek Mountain Range, the Sobaek Mountain Range trends southwest across the center of the peninsula. The peaks of the Sobaek Mountains are also well over 1,000 m above sea level. The Noryeong Mountain Range splits off from the Sobaek Mountains and generally separates the southwestern coastal plains from the western plains in Korea. The southwestern coastal plains have a humid subtropical climate, whereas the western plains have a humid continental climate. These mountain ranges could all be barriers to dispersal of *H. japonica*. Two localities, DY and SC, are located in the southwestern region, and five localities, GN, UJ, AD, CN, and US, are located in the eastern region, east of the Taebaek-Sobaek Mountain ranges. The remaining seven localities are located in the western region of the mainland Korea.

In addition to localities in the mainland, we recorded calls of *H. japonica* at three localities on Jeju Island, which is the largest island in Korea. This island was created entirely from volcanic eruptions approximately 2 million years ago and is dominated by Halla Mountain with a peak of 1,950 m. The vegetation of this island ranges from subtropical forests at the base of the mountain to alpine plants near the peak of the mountain with increasing altitude. J1 was a lawn, and J2 was a pond in the Jejudo Seogipo Natural Recreation Forest that contained trees of humid subtropical and humid continental climates. J3 was a highland wetland. The minimum distance between localities there was 4 km, that is, between J2 and J3 in Jeju Island, whereas the minimum distance between localities on the mainland was 28 km.

### Recordings of advertisement calls

We recorded frog calls between dusk and midnight. All calls were recorded with one of two shotgun microphones (Sennheiser ME64 with K6 powering modules; frequency response ±2.5 dB from 40 to 20,000 Hz; Hanover, Germany), which was placed as close to singing males as possible. Output from a microphone was fed into one of two flash-memory recorders (TASCAM HD-P2, Tokyo, Japan or Sony PCM-D50, Tokyo, Japan) with a 44.1-kHz sampling rate and 16-bit resolution. Calls were recorded for at least one min. One individual frog contributed to only one recording. During each recording night, we recorded the ambient air temperature and relative humidity at the time of the first and last recording using a thermo-hygrometer (Lufft C200; Fellbach, Germany; range: −20 to +50°C; accuracy of temperature measurement: ±0.3°C; accuracy of relative humidity measurement: ±2%), which was placed 10 cm above the ground near the recording sites. We measured air temperature instead of water temperature in this study, because *H. japonica* usually called on the ground or tree branches, not in the water. After recording, we captured the recorded males and put them individually in plastic containers (7×7×4.5 cm) for morphological measurements. We measured the snout-to-vent length (SVL) of each individual, which was defined as the length from the tip of snout to the cloacal opening, to the nearest 0.1 mm, using digital calipers (Mitutoyo; Kanagawa, Japan). For a tissue sample for independent species identification and population genetic structure, we clipped one of the frog's toes using sharp scissors. After toe clipping, antibacterial ointment was applied to the cut toe to prevent infection. The tissue sample was preserved in 90% alcohol. Based on DNA analysis, we verified that all male frogs recorded for this study were *H. japonica*. After morphological measurements and tissue collections were made, we released the captured frogs at their original localities, usually the day after recording.

**Figure 2:**
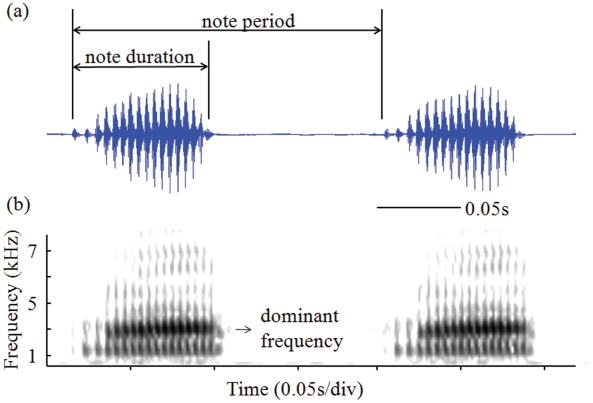
Graphic representation of the advertisement call of *H. japonica*. The call of *H. japonica* consists of a train of notes. The top panel shows an oscillogram of two notes, and the bottom panel is the spectrogram of these two notes. See the text for definitions of call characters.

The advertisement call of *H. japonica* is a train of notes (Fig. 2), each one of which is a unit of sound consisting of one or more pulses (McLister et al. 1995). Twenty-five consecutive notes from each call were analyzed with Raven (version 1.3; Cornell Laboratory of Ornithology, Ithaca, New York, USA). Three call characters were measured: note duration (ND), note repetition rate (NRR), and dominant frequency (DF). ND is the duration of each note from the beginning of the first pulse to the end of the last pulse. NRR is the number of notes per second. We calculated NRR as the inverse of note period, which is defined as the time interval from the beginning of one note to the beginning of the subsequent note. DF was defined as the frequency with the most energy.

Although our analysis of advertisement calls was limited to these three characters, unmeasured characters based on different analytical methods [37], [38], [39] could reveal call characters that may be of important for mate choice in *H. japonica*.

### Statistical analyses for advertisement calls

Geographic variation in body size was analyzed with a one-way analysis of variance (ANOVA) with body size as a response variable and locality as a predictor variable. We did the same analysis twice: first, using all samples, and second, using the mainland samples only, excluding samples from the three Jeju Island localities. A multiple regression analysis was used to understand the effects of spatial variables such as latitude, longitude and elevation on body size.

In Korea, geographic barriers such as mountain ranges and open sea may separate *H. japonica* populations into four groups: western, southwestern, eastern and Jeju Island. The western group included CC, SE, IC, SN, SW, JC and CJ, and the eastern group contained GN, UJ, AD, CN and US. The southwestern group included DY and SC, and the Jeju Island group had three localities, J1, J2, and J3. To examine the effects of geographic barriers on call characters, we used multi-way multivariate analysis of covariance (MANCOVA) with response variables NRR, ND and DF. Barrier and locality were fixed factors, with locality nested within barrier. Covariates were SVL, temperature and humidity. We first tested the assumption of slope parallelism [40]. Three interaction terms, barrier-SVL, barrier-temperature and barrier-humidity, were added to the MANCOVA model that had predictor variables including barrier, locality, SVL, temperature, and humidity. Multiple regressions with stepwise selection were performed to test the effects of spatial variables on call characters.

To test effects of habitat and patry on call characters, we used the residuals of the MANCOVA with predictor variables including barrier, locality, SVL, temperature and humidity. One-way analysis of variance (ANOVA) was performed on residuals for three call characters. Habitat was a predictor variable. In addition, *H. suweonenisis* occurs sympatrically with *H. japonica* in Gyeonggi province (Fig. 1). Likewise, we performed one-way ANOVA on residuals to test the difference in call characters between localities sympatric with *H. suweonensis* (IC, SE, SN, SW) and allopatric localities (CC, JC, CJ). We limited the analysis of patry to the western group only, because some call characters of the western group differed from those of other groups (see the Results). All statistical analyses were performed using SPSS (version 17.0, Chicago, Illinois, USA).

### Population genetics analysis

We used four groups of the tree frog populations (western, eastern, southwestern, Jeju Island) as a priori grouping for the population genetic analysis. The population genetic study was conducted on samples of all but one locality, CJ (Table 1). Because some recordings were discarded for recording analyses due to poor quality, the numbers of recording samples were usually smaller than the numbers of tissue samples for DNA analysis. In two localities, the numbers of tissue samples were larger than the numbers of recording samples, because we were unable to extract DNA for analyses.

Genomic DNA was isolated from the tree frog toe samples using LaboPass™ Tissue Mini kits (Cosmo Genetech Co., Ltd.) following the manufacturer's protocol. A partial fragment of 657 base pairs (bp) from the mitochondrial *COI* (cytochrome oxidase subunit I) gene was amplified by polymerase chain reaction (PCR) using the primer pair LepF1 (5′-ACC AAT CAT AAA GAT ATT GGT-3′ ) and LepR1 (5′-CCT CTG GGT GTC CGA AAA ATC A-3′) [41]. The temperature conditions for PCR were 94°C for 1 min, followed by 35 cycles at 94°C for 40 s, 51°C for 40 s, and 72°C for 60 s, with terminal elongation at 72°C for 5 min. DNA sequencing was performed with the same primers for both directions using an ABI PRISM 3100 automatic sequencer (Applied Biosystem Inc., USA). *COI* sequences were aligned with AlignIR software (LI-COR Biosciences Inc., USA) and then were trimmed manually. A total of 339 *COI* sequences were aligned using ClustalW, implemented with MEGA ver. 4.0 software [42]. DNA sequences were determined and deposited into GenBank (accession HM439113–HM439207). Unique haplotypes among all DNA sequences and the most parsimonious networks among them were analyzed with the computer program TCS [43]. Analysis of molecular variance (AMOVA) for hierarchical population genetic structure and Mantel tests to correlate genetic and geographic distances were performed using the population genetics computer program Arlequin ver. 3.5 [44]. To examine the possibility of recent population expansion, neutrality tests of the population *COI* data were assessed through Tajima's *D* and Fu's *F*s statistics using Arlequin ver. 3.5.

The relationship between geographic distance and genetic distance between localities was tested by the partial Mantel test of matrix association. The geographic distance was calculated as the shortest distance between two localities using geographic coordinates. In addition, we also tested the relationship between geographic distance and difference in call character using partial Mantel tests. To estimate differences in call characters, we used the predicted values of the MANCOVA with barrier, locality, temperature, humidity, and SVL as predictor variables and covariates. The difference in a call character was the absolute difference in values of a call character between two localities. Mantel tests were performed with 1,000,000 permutations for both the full dataset and the mainland-only dataset.

## Results

### Geographic variation in body size

**Figure 3:**
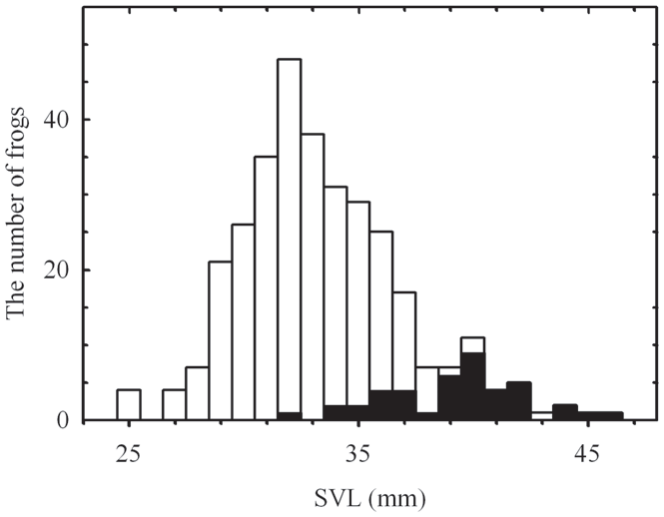
Histogram of SVL of recorded frogs. Snout-Vent Lengths (SVLs) of *H. japonica* from 17 localities in Korea (*n* = 324) are represented. The solid areas denote samples from the three Jeju Island localities (*n* = 42). The bin size is 1 mm.

Body size of *H. japonica* measured as SVL ranged from 25.2 to 46.3 mm with a mean (± SD) of 33.9±3.58 mm. The distribution of SVL deviated from the normal distribution (Kolmogorov-Smirnov with Lilliefors significance correction = 0.078, *P*<0.001, *df* = 324) (Fig. 3). Body size differed significantly between the Jeju Island localities and the mainland localities (*t* = −14.25, *P*<0.0001, *n* = 324). That is, frogs from the three Jeju Island localities were larger than frogs from the 14 mainland localities, making the distribution of SVL skewed to the right (Fig. 3). Because of the possibility that samples from Jeju Island could bias further statistical analyses, we performed all further analyses twice, using two different datasets for analyses of advertisement calls: (1) the full dataset, including all samples and (2) the mainland-only dataset, excluding the Jeju Island samples.

**Figure 4:**
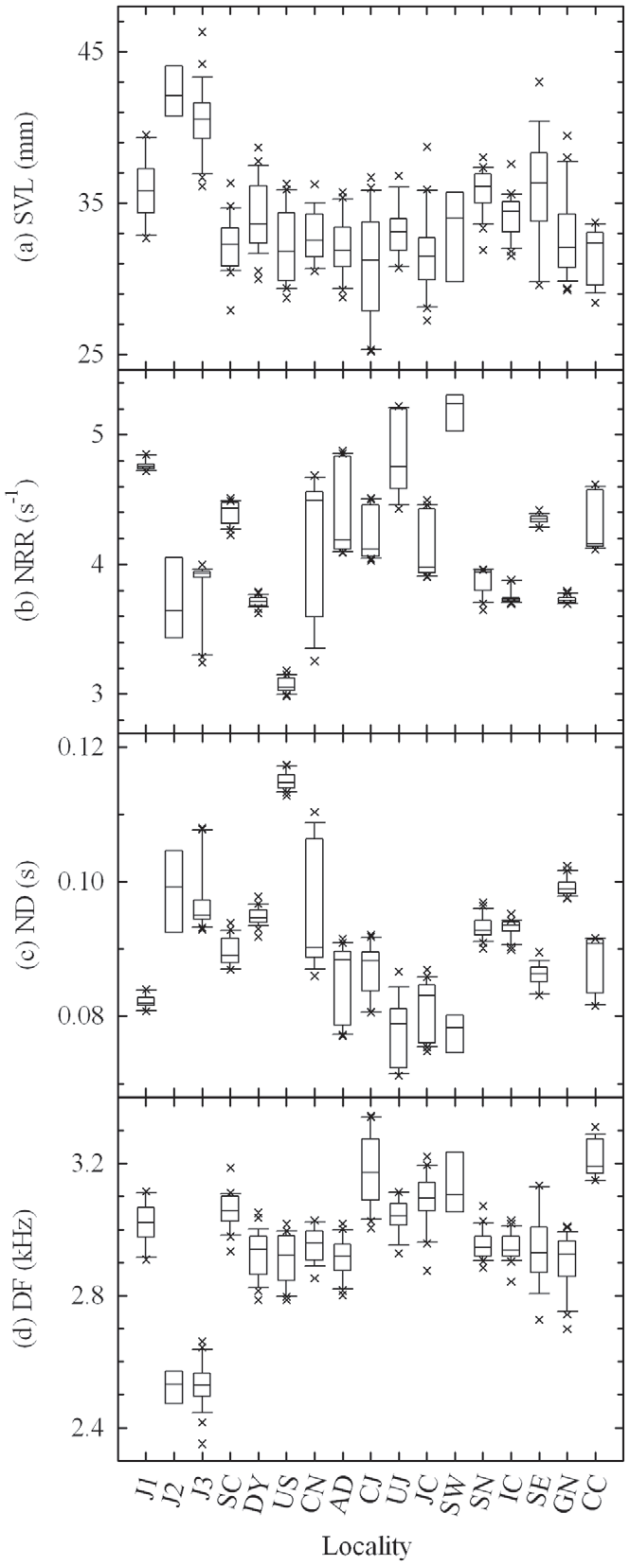
Distributions of body size and call characters. Distributions of body size measured as SVL (a) are grouped into localities. Distributions of NRR (b), ND (c), and DF (d) were regressed against temperature, humidity, and body size; the predictor values of these regression analyses are represented here. Localities are arranged in order of increasing latitude. For each box plot, the line within the box represents the median; the top and bottom lines represent 75th and 25th percentiles, respectively; top and bottom whiskers represent 95th and 5th percentiles, respectively; asterisks represent outliers. Abbreviation and full name of each locality can be found in Table 1. See text for definitions of call characters.

**Figure 5:**
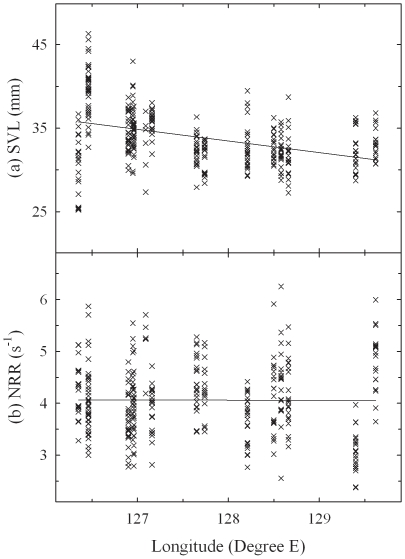
Longitudinal distributions of body size and NRR. Using the full dataset (*n* = 324), the effect of longitude was detected only in body size (a) and NRR (b). The ordinary least squares regression lines are also included in the graphs.

**Figure 6:**
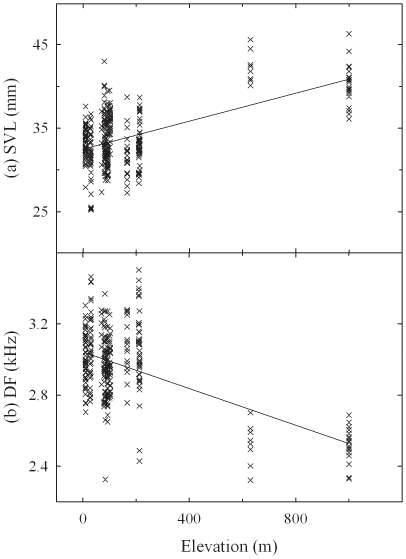
Altitudinal distributions of body size and DF. Using the full dataset (*n* = 324), the effect of altitude was detected only in body size (a) SVL and DF (b). The ordinary least squares regression lines are also included in the graphs.

Using the full dataset, body size significantly differed among localities (one-way ANOVA; *F*_16, 307_ = 28.287, *P*<0.001; Fig. 4a). Instead of locality, we used latitude, longitude and elevation as predictor variables in multiple regressions. SVL (*F*_3, 320_ = 76.427, *P*<0.001; *r* ^2^ = 0.417) was significantly affected by longitude (*β* = −0.264; *t* = −5.584, *P*<0.001; Fig. 5a) and elevation (*β* = 0.461; *t* = 8.104, *P*<0.001; Fig. 6a), but not by latitude (*β* = −0.040; *t* = −0.740, *P* = 0.460). For the mainland-only dataset, locality was still a significant factor for SVL (*F*_13, 268_ = 9.081, *P*<0.001). However, SVL with exclusion of the Jeju Island samples (*F*_3, 278_ = 8.186, *P*<0.001; *r* ^2^ = 0.081) was only affected by longitude (*β* = −0.258; *t* = −4.462, *P*<0.001). Latitude (*β* = 0.104; *t* = 1.806, *P* = 0.072) and elevation (*β* = −0.013; *t* = −0.220, *P* = 0.826) were not significant factors for SVL.

### Geographic variation in advertisement calls

In MANCOVA with interaction terms, none of the three interaction terms were significant: between barrier and SVL (Wilks' λ = 0.978, *F*_9,713_ = 0.711, *P* = 0.699), between barrier and temperature (Wilks' λ = 0.945, *F*_9,713_ = 1.847, *P* = 0.057), and between barrier and humidity (Wilks' λ = 0.976, *F*_9,713_ = 0.784, *P* = 0.631). Thus, the assumption of slope parallelism was met. For the full dataset, the result of the MANCOVA without interaction terms showed that locality was a significant factor for all three call characters, adjusting for SVL, temperature and humidity (Table 2a; Fig. 4b, 4c, 4d). Barrier (Wilks' λ = 0.810, *F*_9,735_ = 7.371, *P*<0.001) was a significant factor for DF, but not for NRR or ND. When SVL was set to 33.93 mm, temperature to 21.58°C and humidity to 93.18%, the estimated marginal means of DF were 3047±15 Hz in the western group, 2903±17 Hz in the eastern group, 2965±22 Hz in the southwestern group, and 2862±30 Hz in the Jeju Island group. There were significant differences in DF for every pair of groups (post hoc comparison with LSD, *P*≤0.041), except between the eastern and Jeju Island groups (*P* = 0.271). Therefore, mountain ranges and open sea may have acted as geographic barriers to the tree frog species. Likewise, for the mainland-only dataset, barrier was (Wilks' λ = 0.889, *F*_6,526_ = 5.326, *P*<0.001) a significant factor for DF, but not for NRR or ND (Table 2b).

**Table 2:** Results of multi-way MANCOVA testing the barrier effect on call characters, NRR, ND and DF.

Source	Character	*df*	Mean Square	*F*	*P*
(a) All samples (*n* = 324)					
Barrier	NRR	3	0.638	2.503	0.059
	ND	3	0.00005	0.790	0.500
	DF	3	336249	19.748	<0.001
Locality(Barrier)	NRR	13	1.587	6.228	<0.001
	ND	13	0.0005	8.554	<0.001
	DF	13	81115	4.764	<0.001
SVL	NRR	1	0.164	0.642	0.423
	ND	1	0.0004	6.229	0.013
	DF	1	1518382	89.174	<0.001
Temperature	NRR	1	14.001	54.943	<0.001
	ND	1	0.004	70.256	<0.001
	DF	1	524	0.031	0.861
Humidity	NRR	1	0.681	2.672	0.103
	ND	1	0.0003	5.288	0.022
	DF	1	356	0.021	0.885
(b) Mainland samples (*n* = 282)					
Barrier	NRR	2	0.535	2.037	0.132
	ND	2	0.00001	0.164	0.849
	DF	2	276850	15.360	<0.001
Locality(Barrier)	NRR	11	1.646	6.264	<0.001
	ND	11	0.0005	9.110	<0.001
	DF	11	50288	2.790	0.002
SVL	NRR	1	0.171	0.652	0.420
	ND	1	0.003	4.815	0.029
	DF	1	1460450	81.028	<0.001
Temperature	NRR	1	12.136	46.174	<0.001
	ND	1	0.004	64.860	<0.001
	DF	1	630.058	0.035	0.852
Humidity	NRR	1	0.601	2.285	0.123
	ND	1	0.0003	5.634	0.018
	DF	1	904.486	0.050	0.823

Geographic barriers included mountain ranges and open sea and divided the populations of *H. japonica* in Korea into four groups: western, eastern, southwestern, and Jeju Island. Locality was nested within barrier. The MANCOVA was conducted on all samples (a) and on the mainland samples only (b).

In the multiple regression analyses using the full dataset, there was no significant clinal variation in ND (Table 3a). NRR was affected by latitude and longitude (Fig. 5b). As longitude increased, male tree frogs produced calls with longer NRR. Elevation was a significant factor for DF (Table 3a, Fig. 6b). As elevation increased, males produced calls with lower DF. In the mainland-only dataset, multiple regression analyses showed that none of the three geographic coordinate factors was significant for ND or DF (Table 3b). However, longitude and elevation affected NRR. Therefore, there seemed to be longitudinal variation in NRR of *H. japonica* in both datasets.

**Table 3:** Results of multiple regressions examining the relationships between call characters of *H. japonica* and spatial variables such as latitude, longitude, and elevation.

Character	Source	*β*	*t*	*P*
(a) All samples (*n* = 324)				
NRR	Latitude	−0.118	−1.975	0.049
	Longitude	0.136	2.312	0.021
	Elevation	−0.074	−1.082	0.280
	Temperature	0.633	12.161	<0.001
	Humidity	0.127	2.359	0.019
	SVL	−0.018	−0.308	0.758
ND	Latitude	−0.048	−0.894	0.372
	Longitude	−0.014	−0.264	0.792
	Elevation	−0.079	−1.288	0.199
	Temperature	−0.702	−15.054	<0.001
	Humidity	−0.169	−3.510	0.001
	SVL	0.188	3.504	0.001
DF	Latitude	0.056	1.203	0.230
	Longitude	−0.088	−1.926	0.055
	Elevation	−0.255	−4.262	<0.001
	Temperature	0.246	6.121	<0.001
	Humidity	0.119	2.860	0.005
	SVL	−0.591	−12.758	<0.001
(b) Mainland samples (*n* = 282)				
NRR	Latitude	−0.087	−1.657	0.099
	Longitude	0.138	2.470	0.014
	Elevation	−0.121	−2.397	0.017
	Temperature	0.663	11.424	<0.001
	Humidity	0.153	2.600	0.010
	SVL	0.003	0.050	0.960
ND	Latitude	−0.016	−0.347	0.729
	Longitude	−0.23	−0.469	0.640
	Elevation	0.006	0.123	0.902
	Temperature	−0.725	−13.946	<0.001
	Humidity	−0.187	−3.555	<0.001
	SVL	0.121	2.647	0.009
DF	Latitude	0.041	0.802	0.423
	Longitude	−0.101	−1.877	0.062
	Elevation	0.052	1.055	0.292
	Temperature	0.301	5.340	<0.001
	Humidity	0.132	2.323	0.021
	SVL	−0.562	−11.372	<0.001

In the all-samples dataset (a), the results of multiple regressions revealed significant variation in NRR (*r^2^* = 0.342; *F*_6, 317_ = 27.508, *P*<0.001), ND (*r^2^* = 0.472; *F*_6, 317_ = 47.320, *P*<0.001), and DF (*r^2^* = 0.607; *F*_6, 317_ = 81.559, *P*<0.001). For the mainland-only dataset excluding the Jeju Island samples (b), multiple regressions showed significant of variation in NRR (*r^2^* = 0.347; *F*_6, 275_ = 24.372, *P*<0.001), ND (*r^2^* = 0.477; *F*_6, 275_ = 41.767 *P*<0.001), and DF (*r^2^* = 0.386; *F*_6, 275_ = 28.845, *P*<0.001). *β* values are standardized coefficients.

Next, we tested whether call characters differed between habitat types. The result of one-way ANOVA on the full dataset showed that habitat was not a significant factor for any call character (Table 4a). Likewise, we tested whether call characters differed between localities sympatric with *H. suweonensis* and allopatric localities in the western group. Patry was not a significant factor for any call character (Table 4b).

**Table 4:** The effects of habitat (a) and patry (b) on call characters, NRR, ND and DF.

		*d.f.*	Mean square	*F*	*P*
(a) Habitat (*n* = 324)					
NRR	Between group	1	0.000	0	1
	Within group	322	0.241		
ND	Between group	1	0.000	0	1
	Within group	322	0.000		
DF	Between group	1	0.000	0	1
	Within group	322	16075		
(b) Patry (*n* = 127)					
NRR	Between group	1	0.000	0	1
	Within group	125	0.251		
ND	Between group	1	0.000	0	1
	Within group	125	0.000		
DF	Between group	1	0.000	0	1
	Within group	125	15951		

We performed a MANCOVA with predictor variables including barrier, locality, SVL, temperature and humidity (see Methods). One-way ANOVA was conducted on residuals of MANCOVA for three call characters. Habitat that was either rice paddy or non-rice paddy was a predictor variable in one-way ANOVA using all samples. Patry was either allopatry or sympatry. Localities only in the western group were included to test the effect of patry.

### Population genetic analyses

We found 95 unique haplotypes of *COI* among all 339 individual tree frogs from 16 localities in Korea. While the average haplotype diversity was very high (*h* = 0.88±0.015), the average nucleotide difference among individuals was very low (*π* = 0.0088±0.0047), falling below the general intraspecific level of divergence. The high haplotype diversity resulted in a complex haplotype network, shaped by several major haplotypes connected by numerous singular hypotypes (Fig. 1). By analyzing the global shape of the network and nucleotide distances among haplotypes and their frequencies, we were able to group the haplotypes into four representative haplogroups (Fig. 1).

Overall, the geographic distribution of the haplogroups appeared to be congruent with the hypothetical grouping of populations into four regions bounded by mountain ranges and sea. Pairwise comparisons of population *COI* sequences supported this congruency. The average values of difference per nucleotide between groups were estimated as follows: between western and eastern (0.010), western and southwestern (0.0134), western and Jeju Island (0.011), eastern and southwestern (0.0093), eastern and Jeju Island (0.0079), and southwestern and Jeju Island (0.0071). The average value among individuals within each population (0.0055) was lower than that between groups (0.0098), indicating low gene flow between populations.

When partitioned into three hierarchical levels according to the four hypothetical groups (western, southwestern, eastern and Jeju Island), the highest proportion of genetic variation resided in the comparison of individuals within populations (50.8%), followed by among groups (35.2%), and was very low between geographically clustered populations within groups (14%) (Table 5). The genetic differentiation test showed statistically significant differentiation among groups (*Φ*_CT_ = 0.35; *P*<0.001) and among populations (*Φ*_ST_ = 0.49; *P* value<0.001). It was also significant among groups (*Φ*_CT_ = 0.33; P<0.001) and among populations (*Φ*_ST_ = 0.46; P<0.001) for the mainland-only dataset, indicating apparent population genetic structures of the tree frog, *H. japonica*, in Korea.

**Table 5:** The results of AMOVA for the hierarchical subdivision of *H. japonica* in Korea, using 657 base pair (bp) of the *COI* gene.

Source of variation	*d.f.*	Sum of squares	% variation
(a) All samples (*n* = 339)			
Among groups	3	314.776	35.21
Among populations within groups	10	119.550	13.98
Within populations	325	543.430	50.81
Total	338	977.755	
(b) Mainland samples only (*n* = 295)			
Among groups	2	219.337	32.72
Among populations within groups	10	116.055	12.82
Within populations	282	523.907	54.47
Total	294	859.298	

Based on mountain ranges and open sea, the populations of *H. japonica* in Korea were divided into four groups: western, eastern, southwestern, and Jeju Island. AMOVAs were conducted twice: once using all 339 samples from 16 localities (a) and once using the mainland samples only (b).

**Figure 7:**
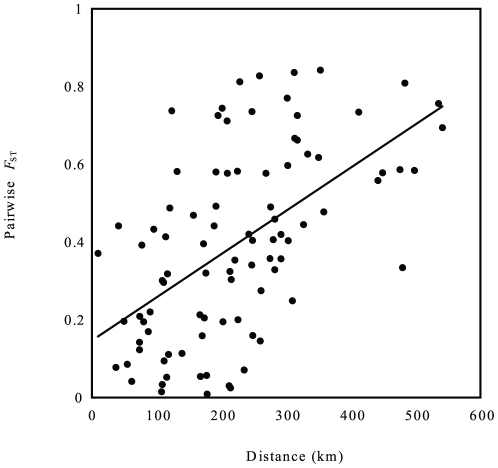
Correlation between genetic and geographic distances. Genetic distance is represented by the estimates of pairwise *F*_ST_ among populations. The regression line was highly significant (*y* = 0.147+0.0011*x; *P*<0.001).

The relationship between genetic and geographic distance showed a statistically very significant correlation both for the full dataset and for the mainland-only dataset (Table 6), indicating IBD in tree frogs within Korea (Fig. 7). However, there were no significant correlations between genetic distance and any of call characters (Table 6). The neutrality of the *COI* sequences was evaluated with two statistical tests, Tajima's *D* and Fu's *Fs*. Both tests revealed statistically significant negative values (Tajima's *D* = −1.54, *P* = 0.021; Fu's *Fs* = −24.68, *P*<0.001), suggesting a possible demographic population expansion of *H. japonica* in Korea during the recent past.

**Table 6:** The relationship between the geographic distance and differences in call characters in *H. japonica*.

	all samples		mainland samples	
	*r*	*P*	*r*	*P*
distance	0.598	<0.001	0.552	<0.001
NRR	−0.072	0.750	−0.151	0.904
ND	0.092	0.178	0.188	0.062
DF	−0.0004	1	−0.384	0.988

Mantel tests were performed for all samples (1,000,000 permutations) and for the mainland samples only (1,000,000 permutations). See the main text for abbreviation of call characters.

## Discussion

### The gene flow hypothesis

The strongest findings for the gene flow hypothesis about geographic variation in call characters were the barrier effect on DF and longitudinal effect on NRR. Major mountain ranges and open sea may explain the geographic variation in DF and NRR of *H. japonica*. The most dominant feature in the geography of the Korean Peninsula is the Taebaek Mountain Range, which runs north and south along the East Sea. During the three-year recording period, we explored the Taebaek Mountains to elevations over 1,000 m to record *H. japonica*, but this effort was not successful. Areas with elevation over 1,000 m in the Taebaek Mountains might not be suitable for *H. japonica*, and it is probably difficult for this species to disperse cross the mountain range. However, we recorded the calls of *H. japonica* in a locality with the elevation over 1,000 m in Jeju Island. Because Jeju Island is located in lower latitudes, its climate is much milder than those of the high ridges in the Taebaek Mountains. Although there is no empirical data about the dispersal ability of *H. japonica*, the Taebaek Mountains may act as a geographic barrier to its dispersal, resulting in longitudinal variation in call characters.

The population genetic structure was largely consistent with the findings of geographic variation in call characters of *H. japonica*. Significant genetic differentiation among groups indicated that mountain ranges and open sea may act as barriers to gene flow in *H. japonica*. Furthermore, the result of significant genetic differentiation among populations was consistent with the pattern of an IBD effect. Because amphibians are generally philopatric [45] and have low dispersal capacity during their lifetimes [46], the restricted gene flow may be the most strong force in shaping the population genetic structure as well as geographic variation in call characters, compared to other evolutionary forces. Therefore, results of both geographic variation in call characters and population genetic structure were consistent with predictions of the gene flow hypothesis in *H. japonica* in Korea.

The tree frog populations in Jeju Island seemed to be distinct from the mainland populations in terms of body size, call characters and population genetics. There was a longitudinal effect on DF for the full dataset, but this effect disappeared for the mainland-only dataset after Jeju Island samples were excluded. In addition, the altitudinal effect on DF and NRR differed between in the full dataset and the mainland-only dataset. The two highest elevations in our study were localities on Jeju Island, and the tree frogs on Jeju Island were generally larger and had lower dominant frequencies than those on the mainland. Thus, a strong altitudinal effect may be responsible for the distinct variation in body size and DF of the tree frog populations in Jeju Island. Being separated since the Pleistocene glacial period, the island and mainland populations may have diverged by vicariance [47]. When a species colonizes an island, it may subsequently diversify to adapt to different ecological niches, eventually producing different species, a process known as adaptive radiation [48]. Sampling should be extended to areas with high elevations in the mainland of Korea and other islands remote from the mainland, because it is not clear whether the samples from Jeju Island were affected by high elevation or isolation from the mainland.

### The selection hypothesis

Although we detected a pattern of IBD in a mitochondrial gene, no such corresponding pattern was observed between geographic distance and call characters. We doubt that lack of correlation between geographic distance and call characters was a result of inadequate sampling, as there was a significant IBD effect on the mitochondrial gene. The effect of IBD might have differently influenced call characters and mitochondrial *COI* sequences in an uncorrelated manner [49], [50]. This conflicting result may also indicate that some selective factors may still be operating on call characters of *H. japonica* in Korea.

Our analyses did not indicate any habitat effect on variation in call characters of *H. japonica* in Korea. However, this study was not designed to systematically examine habitat effect on call characters, and formal studies should be conducted with detailed measurements of habitat characteristics. Properties of acoustic signals are differentially distorted depending on the environment through which the signals are transmitted [51], [52]. Attenuation and reverberation are the two main sources of distortion in temporal and frequency domains of acoustic signals [53], [54]. Even though sound transmission characteristics may be similar across a gradient of adjacent habitats, ambient noise patterns can be dramatically different between habitats [55], [56]. Populations may differentiate in properties of acoustic signals for better transmission or avoidance of acoustic interference in given habitat types along the species' geographic range, causing geographic variation in acoustic signals.

Anthropogenic interference such as noise or pesticide use may influence signal production and transmission in animal communication systems. For example, frogs in a mixed-species assemblage modulated call rates or suppressed calling behaviors in response to airplane flyby or motorcycle noise in central Thailand [57]. Effects of anthropogenic noise on animal communication are reported for birds [58], [59], frogs [57], fish [60], [61], and marine mammals; effects may vary by region depending on the amount and type of anthropogenic noise. In addition, pesticide use has been linked to population decline [62] and developmental anomalies [63], [64], [65] in amphibians. Unfortunately, the possible effects of pesticide use on acoustic communication have not been explored in frogs.

We also found no difference in any call character between areas of sympatry and allopatry in relation to *H. suweonensis*. There is growing evidence that reproductive interaction between closely related species is a selective factor for geographic variation in the communication system of frogs [29], [30], [66], [67]. Comparison of populations between areas of sympatry and allopatry suggests that male signaling traits and female preferences have diverged in a direction to enhance reproductive isolation between sympatric taxa. This pattern of sympatric divergence is termed reproductive character displacement [68], [69], [70]. A possible driving force for the pattern of reproductive character displacement is the reinforcement process in which unfit hybrids are eliminated by natural selection [70], [71], [72]. Recently, the definition of reinforcement has been broadened to include an increase in prezygotic isolation in response to any type of selection against interspecific matings [73]. Although *H. japonica* and *H. suweonensis* are genetically segregated to the level of two separate species [36], [74], reproductive interaction is still possible due to similar morphology and similar call structure [75], [76], [77]. Because the numbers of localities in the sympatry and allopatry datasets were relatively small [78] and because the advertisement calls of *H. suweonensis* were not characterized, it is still premature to conclude that there is no pattern of reproductive character displacement in call characters of *H. japonica*.

Another possible explanation for the lack of relationship between geographic distance and call character is that male advertisement calls may be under intense sexual selection pressure. In 12 populations of a sexually dimorphic damselfly *Calopteryx splendens*, a similar pattern was found: significant IBD for the molecular data, but not for the phenotypic traits [79]. Molecular population divergence was significantly correlated with the population differentiations of eight morphological characters including characteristics of melanized wing patches that were subject to inter-sexual selection [80]. Despite the population structure, measures of selection differentials indicated that population differentiation in morphological characters of *C. splendens* was largely the consequence of interpopulational sexual selection. In many frog species, male advertisement calls are targeted by both intra- and inter-sexual selection [22], [81], [82]. Such pressures of sexual selection may be a strong driving force for differentiation in call characters among populations, exceeding population differentiation predicted by neutral marker loci [83], [84].

### Inference of genetic data

Tajima's *D* and Fu's *F*s tests on population COI data showed a significantly high negative value, indicating sudden population expansion during the recent past. The star-like mtDNA haplotype network (Fig. 1) resembles the typical molecular signature expected under rapid demographic expansion. The Korean Peninsula went through climatic changes during the Pleistocene epoch. The paleoclimatic history in this region would have likely had a profound impact on the evolution and ecology of wild animals and plants. Accordingly, one may expect relevant population genetic signatures reflecting the shrinking and re-opening of suitable habitats followed by demographic and distributional changes in terrestrial animals due to climatic changes. The Quaternary climatic cycles accompanied by glacial and interglacial periods had a profound impact on distribution and evolution of species on a continental scale [85]. Although the impacts of the Pleistocene climatic changes on phylogeography and demography of amphibians have been well addressed in Europe and North and South America [12], research on the effects on amphibians in East Asia remains very sparse. Recently, molecular evidence of rapid population expansion of Black-spotted frogs, *Pelophylax nigromaculatus*, in China was reported [86]. Due to its wide distribution from southernmost China to Japan and Korea, the authors suggested this species as an ideal model for the study of paleoclimatic effects on vertebrates in East Asia. Unfortunately their study did not encompass populations from the Korean Peninsula, so demographic history of *P. nigromaculatus* in this region could not be assessed. Based upon the assumption that the Pleistocene climatic fluctuations had a pervasive influence on historical species distribution in East Asia, the present molecular genetic signature of recent demographic expansion of *H. japonica* provides an additional clue that past glacial events had widespread influence on the evolution and distribution of terrestrial amphibians in East Asia.

In conclusion, studies of genetic diversity using neutral molecular markers show that patterns of isolation by distance or barrier effect may be evident for geographic variation at the molecular level in amphibian species. However, selective factors such as habitat, predator, or interspecific interactions may be critical for geographic variation in sexually selected traits. We studied the effects of gene flow and selection on advertisement calls of *H. japonica*, which is widespread in East Asia. Analyses of field recordings suggested that call characters were largely stratified by geographic distance and by barriers such as mountain ranges and open sea. These findings were consistent with the population genetic structure based on mitochondrial DNA sequences. Although we did not detect effects of habitat or interspecific reproductive interaction, some other selective factors such as sexual selection might be still operating on call characters of *H. japonica* in Korea, in conjunction with restricted gene flow.

## References

[1] Thompson DB, Foster SA, Endler JA (1999). Different spatial scales of natural selection and gene flow: The evolution of behavioral geographic variation and phenotypic plasticity.. Geographic variation in behavior: Perspectives on evolutionary mechanisms.

[2] Slatkin M (1978). Spatial patterns in the distributions of polygenic characters.. J Theor Biol.

[3] Endler JA (1977). Geographic variation, Speciation, and Clines.

[4] Wright S (1938). Size of population and breeding structure in relation to evolution.. Science.

[5] Wright S (1940). Breeding structure of populations in relation to speciation.. Am Nat.

[6] Slatkin M (1993). Isolation by distance in equilibrium and non-equilibrium populations.. Evolution.

[7] Monsen KJ, Blouin MS (2004). Extreme isolation by distance in a montane frog *Rana cascadae*.. Conserv Genet.

[8] Ohta T (1972). Evolutionary rate of cistrons and DNA divergence.. J Mol Evol.

[9] Kimura M (1983). The neutral theory of molecular evolution.

[10] Kimura M (1968). Evolutionary rate at the molecular level.. Nature.

[11] Avise JC (2000). Phylogeography: The history and formation of species.

[12] Zeisset I, Beebee TJC (2008). Amphibian phylogeography: a model for understanding historical aspects of species distributions.. Heredity.

[13] Pröhl H, Koshy RA, Mueller U, Rand AS, Ryan MJ (2006). Geographic variation of genetic and behavioral traits in northern and southern Tungara frogs.. Evolution.

[14] Hitchings SP, Beebee TJ (1997). Genetic substructuring as a result of barriers to gene flow in urban *Rana temporaria* (common frog) populations: implications for biodiversity conservation.. Heredity.

[15] Rowe G, Beebee TJC, Burke T (2000). A microsatellite analysis of natterjack toad, *Bufo calamita*, metapopulations.. Oikos.

[16] Fouquet A, Gilles A, Vences M, Marty C, Blanc M (2007). Underestimation of species richness in neotropical frogs revealed by mtDNA analyses.. PLoS ONE.

[17] Guarnizo CE, Amezquita A, Bermingham E (2009). The relative roles of vicariance versus elevational gradients in the genetic differentiation of the high Andean tree frog, *Dendropsophus labialis*.. Mol Phylogenet Evol.

[18] Bernal XE, Guarnizo C, Lüddecke H (2005). Geographic variation in advertisement call and genetic structure of *Colostethus palmatus* (anura, dendrobatidae) from the Colombian andes.. Herpetologica.

[19] Barber PH (1999). Patterns of gene flow and population genetic structure in the canyon treefrog, *Hyla arenicolor* (Cope).. Mol Ecol.

[20] Barber PH (1999). Phylogeography of the canyon treefrog, *Hyla arenicolor* (Cope) based on mitochondrial DNA sequence data.. Mol Ecol.

[21] Funk WC, Blouin MS, Corn PS, Maxell BA, Pilliod DS (2005). Population structure of Columbia spotted frogs (*Rana luteiventris*) is strongly affected by the landscape.. Mol Ecol.

[22] Gerhardt HC, Huber F (2002). Acoustic communication in insects and anurans.

[23] Ryan MJ, Rand AS, Weigt LA (1996). Allozyme and advertisement call variation in the Tungara frog, *Physalaemus pustulosus*.. Evolution.

[24] Wycherley J, Doran S, Beebee TJC (2002). Frog calls echo microsatellite phylogeography in the European pool frog (*Rana lessonae*).. J Zool (Lond).

[25] Boul KE, Funk WC, Darst CR, Cannatella DC, Ryan MJ (2007). Sexual selection drives speciation in an Amazonian frog.. Proc Roy Soc B.

[26] Bosch J, De La Riva I (2004). Are frog calls modulated by the environment? An analysis with anuran species from Bolivia.. Can J Zool.

[27] Kime NM, Turner WR, Ryan MJ (2000). The transmission of advertisement calls in Central American frogs.. Behav Ecol.

[28] Pröhl H, Ron SR, Ryan MJ (2010). Ecological and genetic divergence between two lineages of Middle American tungara frogs *Physalaemus* ( = *Engystomops*) *pustulosus*.. BMC Evol Biol.

[29] Gerhardt HC (1994). Reproductive character displacement of female mate choice in the grey treefrog, *Hyla chrysoscelis*.. Anim Behav.

[30] Höbel G, Gerhardt HC (2003). Reproductive character displacement in the acoustic communication system of green tree frogs (*Hyla cinerea*).. Evolution.

[31] Fouquette MJJ (1975). Speciation in chorus frogs. I. Reproductive character displacement in the *Pseudacris nigrita* complex.. Syst Zool.

[32] Animal Behviour Society (2006). Guidelines for the treatment of animals in behavioural research and teaching.. Anim Behav.

[33] Kuramoto M (1980). Mating calls of treefrogs (genus *Hyla*) in the Far East, with description of a new species from Korea.. Copeia.

[34] Yang S-Y, Park B-S (1988). Speciation of the two species of the genus *Hyla* (Anura) in Korea.. Kor J Zool.

[35] Yang S-Y, Min M-S (1997). Intra and interspecific diversity and speciation of two tree frogs in the genus *Hyla*.. Kor J Genet.

[36] Lee JE, Yang DE, Kim YR, Lee H, Lee HI (1999). Genetic relationships of Korean treefrogs (Amphibia; Hylidae) based on mitochondrial cytochrome b and 12S rRNA genes.. Kor J Biol Sci.

[37] Benko TP, Perc M (2006). Deterministic chaos in sounds of Asian cicadas.. J Biol Syst.

[38] Benko TP, Perc M (2007). Singing of Neoconocephalus robustus as an example of deterministic chaos in insects.. J Biosci.

[39] Kodba S, Perc M, Marhl M (2005). Detectic chaos from a time series.. Eur J Phys.

[40] Sokal RR, Rohlf FJ (1995). Biometry.

[41] Hebert PDN, Penton EH, Burns JM, Janzen DH, Hallwachs W (2004). Ten species in one: DNA barcoding reveals cryptic species in the neotropical skipper butterfly *Astraptes fulgerator*.. Proc Natl Acad Sci USA.

[42] Kumar S, Tamura K, Nei M (2004). MEGA3: Integrated software for molecular evolutionary genetics analysis and sequence alignment.. Brief Bioinform.

[43] Clement M, Posada D, Crandall KA (2000). TCS: a computer program to estimate gene genealogies.. Mol Ecol.

[44] Excoffier L, Lischer HEL (2010). Arlequin suite ver 3.5: a new series of programs to perform population genetics analyses under Linux and Windows.. Mol Ecol Resour.

[45] Waldmann B, McKinnon JS (1993). Inbreeding and outbreeding in fishes, amphibians and reptiles.

[46] Stebbins RC, Cohen NW (1995). A natural history of amphibians.

[47] Lefebvre T, Chaline N, Limousin D, Dupont S, Bagneres A-G (2008). From speciation to introgressive hybridization: the phylogeographic structure of an island subspecies of termite, *Reticulitermes lucifugus corsicus*.. BMC Evol Biol.

[48] Schluter D (2000). The ecology of adaptive radiation.

[49] Cuenca A, Escalante AE, Piñero D (2003). Long-distance colonization, isolation by distance, and historical demography in a relictual Mexican pinyon pine (*Pinus nelsonii* Shaw) as revealed by paternally inherited genetic markers (cpSSRs).. Mol Ecol.

[50] Knoll S, Rowell-Rahier M (1998). Distribution of genetic variance and isolation by distance in two leaf beetle species: *Oreina cacaliae* and *Oreina speciosissima*.. Heredity.

[51] Ryan MJ, Kime NM, Simmons AM, Popper AN, Fay RR (2002). Selection on long-distance acoustic signals.. Acoustic communication.

[52] Bradbury JW, Vehrencamp SL (1998). Principles of Animal Communication.

[53] van Dongen WFD, Mulder RA (2006). Habitat density, song structure and dialects in the Madagascar paradise flycatcher *Terpsiphone mutata*.. J Avian Biol.

[54] Doutrelant C, Leitao A, Giorgi M, Lambrechts MM (1999). Geographic variation in blue tit song, the result of an adjustment to vegetation type?. Behaviour.

[55] Slabbekoorn H (2004). Habitat-dependent ambient noise: Consistent spectral profiles in two African forest types.. J Acoust Soc Am.

[56] Slabbekoorn H, Smith TB (2002). Habitat-dependent song divergence in the little greenbul: An analysis of environmental selection pressures on acoustic signals.. Evolution.

[57] Sun JWC, Narins PM (2005). Anthropogenic sounds differentially affect amphibian call rate.. Biol Conserv.

[58] Wood WE, Yezerinac SM, Dufty J, A. M. (2006). Song sparrow (*Melospiza melodia*) song varies with urban noise.. Auk.

[59] Slabbekoorn H, Ripmeester EAP (2008). Birdsong and anthropogenic noise: implications and applications for conservation.. Mol Ecol.

[60] Popper AN, Hastings MC (2009). The effects of anthropogenic sources of sound on fishes.. J Fish Biol.

[61] McCauley RD, Fewtrell J, Popper AN (2003). High intensity anthropogenic sound damages fish ears.. J Acoust Soc Am.

[62] Davidson C, Shaffer HB, Jennings MR (2002). Spatial tests of the pesticide drift, habitat destruction, UV-B, and climate-change hypotheses for California amphibian declines.. Conserv Biol.

[63] Carr JA, Gentles A, Smith EE, Goleman WL, Urquidi LJ (2003). Response of larval *Xenopus laevis* to atrazine: Assessment of growth, metamorphosis, and gonadal and laryngeal morphology.. Environ Toxicol Chem.

[64] Hayes T, Haston K, Tsui M, Hoang A, Haeffele C (2003). Atrazine-induced hermaphroditism at 0.1 ppb in American leopard frogs (*Rana pipiens*): laboratory and field evidence.. Environ Health Perspect.

[65] Tavera-Mendoza L, Ruby S, Brousseau P, Fournier M, Cyr D (2002). Response of the amphibian tadpole (*Xenopus laevis*) to atrazine during sexual differentiation of the testis.. Environ Toxicol Chem.

[66] Loftus-Hills JJ, Littlejohn MJ (1992). Reinforcement and reproductive character displacement in *Gastrophryne carolinensis* and *Gastrophryne olivacea* (Anura: Microhylidae): A reexamination.. Evolution.

[67] Marquez R, Bosch J (1997). Male advertisement call and female preference in sympatric and allopatric midwife toads.. Anim Behav.

[68] Brown WL, Wilson EO (1956). Character displacement.. Syst Zool.

[69] Grant PR (1972). Convergent and divergent character displacement.. Biol J Linn Soc.

[70] Howard DS, Harrison RG (1993). Reinforcement: origin, dynamics, and fate of an evolutionary hypothesis.. Hybrid zones and the evolutionary process.

[71] Butlin R (1987). Speciation by reinforcement.. Trends Ecol Evol.

[72] Dobzhansky T (1940). Speciation as a stage in evolutionary divergence.. Am Nat.

[73] Servedio MR, Noor MAF (2003). The role of reinforcement in speciation: theory and data.. Annu Rev Ecol Evol Syst.

[74] Kuramoto M (1984). Systematic implications of hybridization experiments with some Eurasian treefrogs (Genus *Hyla*).. Copeia.

[75] Jang Y, Gerhardt HC (2006). Divergence in female calling song discrimination between sympatric and allopatric populations of the southern wood cricket *Gryllus fultoni* (Orthoptera: Gryllidae).. Behav Ecol Sociobiol.

[76] Jang Y, Gerhardt HC (2006). Divergence in the calling songs between sympatric and allopatric populations of the southern wood cricket *Gryllus fultoni* (Orthoptera: Gryllidae).. J Evol Biol.

[77] Jang Y, Gerhardt HC (2007). Temperature effects on the temporal properties of calling songs in the crickets *Gryllus fultoni* and *G. vernalis*: Implications for reproductive isolation in sympatric populations.. J Insect Behav.

[78] Gabor CR, Ryan MJ (2001). Geographical variation in reproductive character displacement in mate choice by male sailfin mollies.. Proc Roy Soc B.

[79] Svensson EI, Kristoffersen L, Oskarsson K, Bensch S (2004). Molecular population divergence and sexual selection on morphology in the banded demoiselle (*Calopteryx splendens*).. Heredity.

[80] Siva-Jothy M (1999). Male wing pigmentation may affect reproductive success via female choice in a Calopterygid damselfly (Zygoptera).. Behaviour.

[81] Ryan MJ, Keddy-Hector A (1992). Directional patterns of female mate choice and the role of sensory biases.. Am Nat.

[82] Gerhardt HC (1994). The evolution of vocalization in frogs and toads.. Annu Rev Ecol Syst.

[83] Merilä J, Crnokrak P (2001). Comparison of genetic differentiation at marker loci and quantitative traits.. J Evol Biol.

[84] Palo JU, Schmeller DS, Laurila A, Primmer CR, Kuzmin SL (2004). High degree of population subdivision in a widespread amphibian.. Mol Ecol.

[85] Schmitt T (2007). Molecular biogeography of Europe: Pleistocene cycles and postglacial trends.. Front Zool.

[86] Zhang H, Yan J, Zhang G, Zhou K (2008). Phylogeography and demographic history of Chinese black-spotted frog populations (*Pelophylax nigromaculata*): Evidence for independent refugia expansion and secondary contact.. BMC Evol Biol.

[87] Yang S-Y (2000). Monograph of Korean Amphibia.

